# Effects of Intensive Crew Training on Individual and Collective Characteristics of Oar Movement in Rowing as a Coxless Pair

**DOI:** 10.3389/fpsyg.2017.01139

**Published:** 2017-07-06

**Authors:** Mathieu Feigean, Mehdi R’Kiouak, Reinoud J. Bootsma, Jérôme Bourbousson

**Affiliations:** ^1^Institute of Sport Science, University of BernBern, Switzerland; ^2^Movement, Interactions, Performance EA 4334, Faculty of Sport Sciences, University of NantesNantes, France; ^3^Institut des Sciences du Mouvement, Aix-Marseille Université, Centre National de la Recherche ScientifiqueMarseille, France

**Keywords:** joint action, rowing, synchrony, crew behavior, individual pattern

## Abstract

This case study examined how two rowers adapted their rowing patterns following crew training as a newly formed coxless pair. The two participants were expert (double-oar) single scull-boat rowers. Performing as a crew in the coxless-pair’s sweep-boat, where each rower operates a single oar, on-the-water data were collected before and after a 6-week intensive team-training program. Rowing patterns were characterized by the horizontal oar angle, oar angular velocity and linear oar-water velocity profiles during the catch (minimal oar angle) to finish (maximal oar angle) half-cycles of the propulsive water phase. After crew training, rowers demonstrated a tighter synchronization and a closer correspondence in oar angle at the moment of catch, together with a closer matching of the evolution over time of their subsequent oar movements. Most likely due to the inherent asymmetries involved in sweep-boat rowing, the stroke rower also developed a somewhat longer-duration larger-amplitude oar movement than the bow rower. Remarkably, both rowers revealed changes in the inter-cycle variability of their individual patterns of rowing. While the initially more variable stroke rower improved the consistency of his rowing pattern over practice, the initially highly consistent bow rower on the contrary relaxed his tendency to always perform in the same way. We discuss how the crew performance changed over training and to what extent it was associated with changes in individual behaviors. Along the way we demonstrate that the often-used measure of average continuous relative phase does not adequately capture the particularities of the coordination pattern observed. Overall, the results obtained at the individual level of analysis suggest that team benefits were obtained through distinct adaptations of the rowers’ individual rowing patterns.

## Introduction

Joint action is considered as a form of social interaction whereby individual agents coordinate their movement in space and time so as to reach a common goal (Sebanz et al., 2006). While a considerable amount of research has focused on the nature and stability characteristics of coordinative states resulting from informational coupling between individual agents (see Schmidt and Richardson, 2008, for an overview), the tasks considered generally did not have a specific supra-coordinative goal. On the other hand, in tasks like dyadic manual precision aiming, where one participant controls the position of a pointer and another participant controls the position of a target (in the discrete task version, Romero et al., 2015) or a set of two targets (in the reciprocal task version, Mottet et al., 2001), the supra-coordinative goal to have the pointer coincide with the target(s) naturally structures the required between-participant coordination. Focusing on variance in the upper-limb joint angles, Romero et al. (2015) indeed demonstrated that inter-personal synergies were stronger than intra-personal synergies, while Mottet et al. (2001) demonstrated between-participant compensatory variability at the level of the two end-effectors (i.e., the control of the positions of the pointer and target-set).

More generally, in joint action tasks the individual agents’ movements are shaped both by the current needs of their collective behavior and by the singular task demands that each individual agent faces. In this light, expertise in collective behavior tasks has been considered as the capability of individual agents to identify and achieve a specific contribution (e.g., Duarte et al., 2012; Benerink et al., 2016), thus reflecting a coordination of labor within the social joint-action system. Embedded in a process of compensatory variability between individual agents, the collective behavioral states may be expected to depend on the individual agents’ abilities to adapt their own intrinsic behavioral dynamics to the needs of the cooperative effort. In order to characterize such adaptations at the level of the individual agents, in the present study we examined how a pair of rowers adapted their contribution to the joint action task of moving the boat forward after having followed an intensive crew-training (CT) program. By selecting a newly formed crew pair of expert rowers, the present study moreover provided an optimal framework for addressing task-goal driven adaptations in individual behavior in a real-life joint-action task.

**FIGURE 1:**
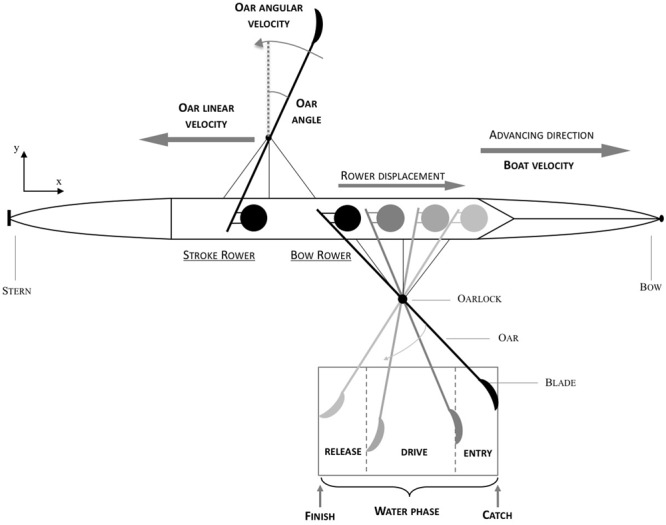
Bird’s-eye view of a coxless pair rowing situation. The water phase of oar movement is delineated by catch (minimal oar angle) and finish (maximal oar angle) points and consists of three parts, the entry, the drive, and the release.

In competitive crew rowing the individual rowers need to coordinate their actions in order to move the boat forward as fast and as efficiently as possible. Perfect synchronization of propulsive oar movement has often been cited as being a prime requirement for efficient rowing (e.g., Wing and Woodburn, 1995; Baudouin and Hawkins, 2004; de Brouwer et al., 2013; Cuijpers et al., 2015, 2016; Seifert et al., 2017). It is important to realize, however, that such a requirement cannot be indistinguishably applied to the two different types of boats used in competitive rowing. In sculling each rower simultaneously operates a pair of oars (one on the left and one on the right) and boats for (crew) sculling are therefore symmetrically rigged. In sweep-oar rowing, on the other hand, each rower operates a single oar (either on the left or on the right) and sweep-oar boats are therefore asymmetrically rigged. In sweep-oar rowing as a coxless pair, as studied in the present contribution, the crew consists of two rowers, with the bow rower being closest to the bow and the stroke rower being closest to the stern (see **Figure 1** for further details). In such a setting, perfect synchronization of oar movement, with its associated symmetrical power output, in fact results in yawing (resulting in changing direction) of the boat during each propulsive drive phase, thereby reducing overall efficiency (Hill, 2002; Barrow, 2010). Well-trained crews may thus be expected to have developed strategies to overcome this (Hill, 2002), while at the same time incorporating the inherently different roles resulting from the in-line placement (i.e., one behind the other) of the individual rowers (in both scull and sweep-oar boats). Indeed, as a result of such in-line placement, the stroke rower is not able to directly see his/her teammate(s). Because there is no cox (short for coxswain, an oar-less crew-member responsible for steering and race strategy), rowing as a coxless pair is self-paced. It is typically the stroke rower that is in charge of setting the rhythm, thereby potentially giving rise to leader-follower roles within the crew (Seifert et al., 2017).

In this light, we investigated how changes in the individual rowing behaviors of a coxless pair were observable over a large time span (i.e., across 6 weeks), after participants had been involved in repeated crew coordination practices. The investigation started from the very first step of their crew training. It ended after a one-month-and-an-half intensive team practice focused on enhancing their coordinative capability. The goal of the study was to simultaneously characterize the changes in the collective and individual rower behaviors.

## Materials and Methods

### Participants and Procedure

Two 17-year-old men participated in the study. Having been admitted into the French National Rowing School (*Pôle Espoir Aviron – Nantes*), both were qualified as expert-level individual rowers. Each rower had more than 10 years of experience in single scull (two-oar) rowing. Rowers individually performed in the national competition and belonged to the French top 10. While both had rowed in crew boats during training sessions, neither had experienced dedicated crew training. Before engaging in the present study they had never rowed together in the same boat.

Data were collected during two on-water rowing sessions as a coxless pair (i.e., in an asymmetrically rigged sweep-oar boat where each rower operates one oar) that took place before and after a 6-week training program dedicated to crew rowing. We will refer to these two data-collection sessions as pre-CT and post-CT, respectively. The intensive CT program was managed by the national coach and comprised 26 (i.e., 4+ per week) on-water practice sessions, for a total of almost 50 h of coxless pair rowing practice. Each practice session typically consisted of two sets of 20–30 min of rowing separated by 5-min rest periods. During training sets, performed at frequencies of 17–28 strokes per minute (spm), rowers had to use maximal power during the drive (i.e., when the oar was in the water), so as to move the boat forward as fast as possible, and to recover when the oar was out of the water. During practice sessions, the coach followed the coxless pair in a motorboat, providing online feedback mainly focusing on the simultaneity of the oars’ entry into the water, the orientation of the blades and the direction of the boat. Crew briefings providing further information were organized before and after each CT training session.

The pre-CT and post-CT data-collection sessions took place under calm water and stable weather conditions while rowing at constant pace of 17–18 spm under the same general instructions as described for the training sessions. Both rowers had extensive previous individual practice experience at this stroke rate. Moreover, it did not induce a level of fatigue that could be expected to alter the rowing patterns over the course of the approximately 20-min sessions during which data was collected.

The study was performed in accordance with the Declaration of Helsinki and the APA ethical guidelines. It was approved by an Institutional Review Board of the University of Nantes. The two rowers and their coaches were informed of the procedures. The rowers, their parents and the staff members in charge provided written informed consent. Both sessions analyzed in the present study were part of a larger research project (ANOPACy), also including qualitative phenomenological analyses of the experience of crew rowing, using individual rower verbalizations obtained during video-based self-confrontation interviews (R’Kiouak et al., 2016) and other rowers and rowing conditions (Seifert et al., 2017).

### Data Collection and Analysis

During the pre-CT and post-CT sessions, behavioral data were collected at 50 Hz using the PowerLine system (Peach Innovations, Cambridge, United Kingdom). For the present purposes, we retained the time series of horizontal oar angles (delivered by position sensors in the oarlocks) and boat velocity (delivered by an impeller fixed under the shell). According to Coker (2010), the PowerLine angle sensors provide an accuracy of 0.5°. No accuracy data are available with respect to boat velocity measurements. For each session, the first 350 recorded strokes were retained for analysis.

**FIGURE 2:**
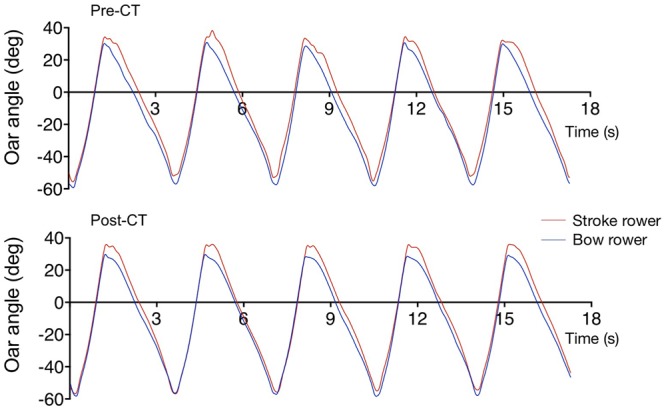
Samples of oar movement of the stroke (red) and bow (blue) rowers for five subsequent strokes during the pre-CT **(Upper)** and post-CT **(Lower)** sessions.

Full time series of the 350 recorded strokes were first filtered using a low-pass Butterworth filter with a 7-Hz cut-off frequency, run through twice in order to negate the phase shift. Oar angular velocity (OAV) time series were subsequently derived using the first central difference method. The first 10 cycles were then removed in order to focus on stabilized performance, leaving 340 full strokes for analysis purposes. Samples of five subsequent strokes from the pre-CT and post-CT sessions are presented in **Figure 2**.

With overall crew performance quantified by boat velocity, data analysis focused on the collective level of between-rower coordination and the individual kinematic level of oar-movement patterns. At the individual rower level, different cycles of oar movement were identified by their catch and finish points, corresponding to the minimum and maximum oar angles (with zero defined as perpendicular to the boat, negative in the direction of the bow and positive in the direction of the stern) for each rower separately. Starting from the catch point, a rower’s full stroke is defined by four subsequent phases: *entry* (where the blade enters the water), *drive* (where the blade drives the boat forward), *release* (where the blade exits the water) ending at the finish point, and *recovery* from the finish to the next catch point (Coker, 2010, p. 45).

In order to quantify individual rower behavior, we extracted for each rower at each session the time series of oar angle and OAV of the half-cycles between catch and finish points. Each of these 340 half-cycles was time-normalized using steps of 2% half-cycle duration, resulting in 51 points per half-cycle. Average time-normalized half-cycles for each rower at each session were then obtained for oar angle and OAV by calculating the mean of all 340 corresponding values at each of the 51 points. The variability over half-cycles was calculated as the standard deviation over all 340 corresponding values at each of the 51 points.

As we were mainly interested in the (propulsive) drive phase, we identified this phase by determining when the oar moved faster than the water. To this end, for each of the 340 extracted half-cycles of each rower in each session, we determined the linear oar velocity in the direction of the boat’s longitudinal axis by multiplying the tangential oar velocity (defined by the product of OAV and oar length) with the cosine of the oar angle. By calculating the difference between instantaneous linear oar velocity and instantaneous boat velocity and averaging over the 340 cycles, we obtained average time-normalized half-cycles of linear oar velocity with respect to the water [linear oar-water velocity (OWV)].

**FIGURE 3:**
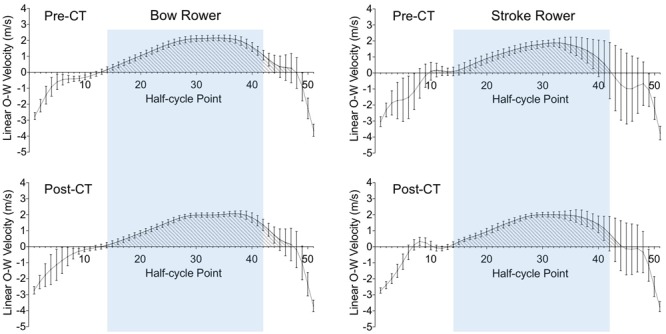
Average time-normalized half-cycles for oar-water velocity from catch (point 1) to finish (point 51) for the bow (Left) and stroke (Right) rowers during the pre-CT (Upper) and post-CT (Lower) sessions. Error bars present the standard deviations over 340 half-cycles. The shaded area delineates the drive phase during which oar-water velocity is positive. The blue area indicates the common part of the drive phase (between points 14 and 42).

As can be seen from **Figure 3**, the drive phase (shaded areas under the curves where linear OWV is positive) ended closer to the finish point for the bow rower than for the stroke rower, at least during the pre-CT session. In order to compare the behavior of individual rowers on and between sessions, for both rowers we therefore selected the common (29-point) period from point 14 to point 42 (i.e., from 26 to 82% of the duration of the catch-finish half-cycle) for analyses of the drive phase.

For the analysis of individual rower behavior, oar angle kinematics were thus determined between the catch and finish points of each individual rower’s actions (i.e., on separated time series). In examining the resultants plots (e.g., **Figure 3**) it is important to realize that these individual catch and finish points did not necessarily coincide in time, as becomes clear from inspection of **Figure 2**. In order to capture the collective behavior of the two rowers, we therefore extracted the 340 synchronous oar angle and OAV time series of the rowers during each (catch-to-finish) half-cycle of the stroke rower. After time-normalization to 51 points according to the procedure described above, for each of these 340 time-locked series we quantified between-rower coordination by the continuous relative phase (CRP) between the motions of the two oars during the catch-finish half-cycles. To this end, for each rower and each half-cycle, oar angle was normalized to a [-1;+1] interval based on the minimal and maximal oar angles (i.e., amplitude normalized) and OAV was normalized by dividing OAV by peak OAV (resulting in a maximum of +1); Phase was determined as the angle (clockwise notation) formed by each point thus defined in the normalized phase plane. CRP was defined at each point as the difference between the phases of the stroke and bow rowers (de Brouwer et al., 2013; de Poel et al., 2016; Seifert et al., 2017). A global measure of rower synchronization during the drive phase was obtained by calculating for each half-cycle the average CRP value over the period between points 14 and 42. This procedure thus resulted in 340 CRP values per session, with a measure of synchronization being provided by the mean and a measure of its variability being provided by the standard deviation over the 340 values. A complementary measure of space-time similarity of oar movement was obtained by calculating the root mean square (RMS) difference of the time-locked oar-angle time series of the two rowers during these same periods.

Statistical comparisons of means based on *n* = 340 observations were performed using independent-sample *t*-tests. Statistical comparisons of variability (defined as standard deviations over 340 observations) were performed using *t*-tests over the 29 points defining the drive phase. Paired tests were used for within-rower comparisons (pre-CT vs. post-CT for bow and for stroke) while independent sample tests were used for between-rower comparisons (bow vs. stroke at pre-CT and at post-CT). Because only one pair of rowers was considered in the present study, significant (α = 0.05) effects were only considered when effect size (Cohen’s *d*) reached at least the 0.50 threshold for a medium size effect (Cohen, 1988). Since, given the number of observations, any effect with effect size *d* ≥ 0.5 was also statistically significant, we only reported *d*-values so as to stress substantive rather than statistical significance.

## Results

During the pre-CT and post-CT sessions rowers demonstrated stroke frequencies of 17.94 ± 0.46 and 17.37 ± 0.28 spm, respectively. The slightly (3.3%) lower stroke rate during the post-CT session was accompanied by a 2.2% lower average boat velocity (pre-CT 3.40 ± 0.08 m/s, post-CT 3.33 ± 0.09 m/s).

As can be already be seen in **Figure 2**, durations of catch-to-finish half-cycles were shorter than durations of the complementary finish-to-catch (recovery) half-cycles. During the pre-CT session the durations of the catch-to-finish half-cycles were 1.016 ± 0.046 s and 1.152 ± 0.111 s for the bow and stroke rower, respectively; during the post-CT session the corresponding durations were 1.057 ± 0.053 s and 1.118 ± 0.073 s, respectively. The difference in (catch-to-finish) half-cycle durations thus decreased over practice (from 0.137 to 0.061 s), but remained significant at the time of the post-CT session, *d* = 0.69.

While amplitudes of oar displacement from catch to finish were almost identical during the pre-CT session (bow 88.3 ± 1.6°; stroke 88.8 ± 1.8°), a difference came to the fore during the post-CT session, mainly due to an increase in amplitude for the stroke rower (bow 87.3 ± 1.3°, stroke 92.9 ± 1.3°; *d* = 3.46. During the pre-CT session peak angular velocity was slightly lower for the stroke rower (115.1 ± 3.3 deg/s) than for the bow rower (117.9 ± 3.3 deg/s), *d* = 0.81. This difference no longer existed during the post-CT session (bow 117.2 ± 3.1 deg/s; stroke 117.2 ± 3.6 deg/s; *d* = 0.01).

**FIGURE 4:**
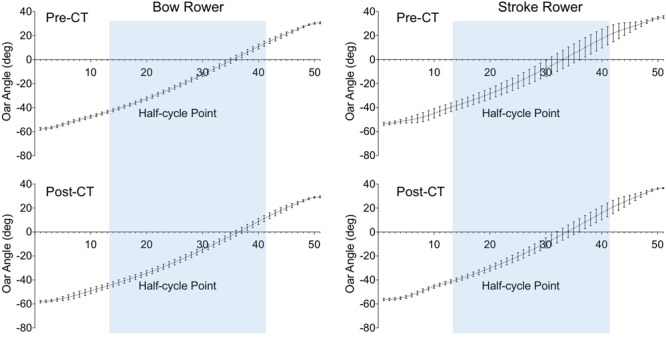
Average time-normalized half-cycles for oar angle from catch (point 1) to finish (point 51) for the bow (Left) and stroke (Right) rowers during the pre-CT (Upper) and post-CT (Lower) sessions. Error bars present the standard deviations over 340 half-cycles. The blue area indicates the common part of the drive phase (between points 14 and 42).

As can be seen from **Figure 4** (error bars), during the pre-CT session the bow rower demonstrated a particularly consistent pattern of oar angular (OA) displacement during the drive phase, with an OA variability (defined as the 29-point average of standard deviations over the 340 drives) of 1.80 ± 0.31°. The stroke rower’s movements during this pre-CT session were considerably more variable (*d* = 3.81), with an OA variability of 6.15 ± 1.59°. Interestingly, over practice not only the stroke rower’s OA variability decreased (post-CT 3.88 ± 1.45°, *d* = 8.31), but the bow rower’s OA variability increased (post-CT 2.34 ± 0.13°, *d* = 2.94). While the difference between individual rower OA variabilities thus decreased over practice, it remained significantly lower for the bow rower, *d* = 1.49.

**FIGURE 5:**
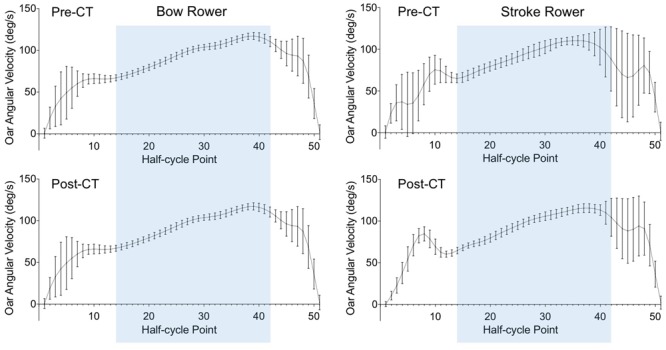
Average time-normalized half-cycles for oar angular velocity from catch (point 1) to finish (point 51) for the bow (Left) and stroke (Right) rowers during the pre-CT (Upper) and post-CT (Lower) sessions. Error bars present the standard deviations over 340 half-cycles. The blue area indicates the common part of the drive phase (between points 14 and 42).

A slightly different pattern of results emerged for the variability in OAV (**Figure 5**) during the drive phase. During the pre-CT session, the OAV variability was smaller for the bow rower (4.75 ± 0.40°) than for the stroke rower (9.03 ± 7.81°), *d* = 0.77. Both rowers decreased their OAV variability over practice, reaching 4.01 ± 0.59° for the bow rower (*d* = 1.35) and 5.68 ± 3.75° for the stroke rower (*d* = 0.73) during the post-CT session. Although the difference between individual OAV variabilities decreased over practice, it remained significantly lower for the bow rower, *d* = 0.62.

Applying the same analysis to the linear OWV (**Figure 3**) revealed a smaller OWV variability for the bow rower (0.21 ± 0.04 m/s) than for the stroke rower (0.47 ± 0.39 m/s) during the pre-CT session, *d* = 0.93. Both rowers decreased their OWV variability over practice, reaching 0.18 ± 0.05 m/s for the bow rower (*d* = 0.95) and 0.28 ± 0.21 m/s for the stroke rower (*d* = 0.99) during the post-CT session. Although the difference between individual OAV variabilities decreased over practice, it remained significantly lower for the bow rower, *d* = 0.63.

**FIGURE 6:**
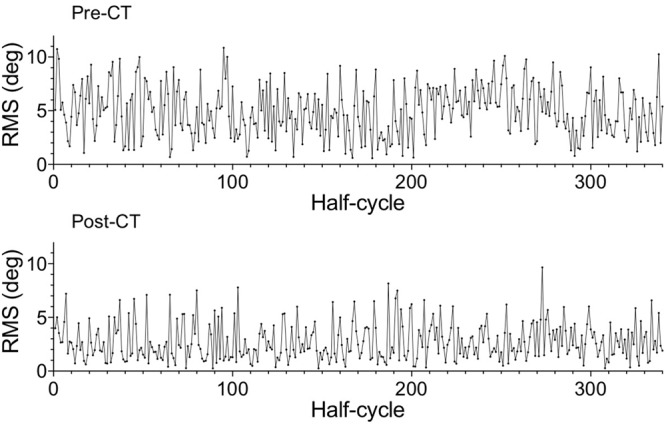
Root mean square (RMS) of the difference between oar angles of the stroke and bow rowers for the 340 catch-finish half-cycles of the pre-CT (Upper) and the post-CT (Lower) sessions.

**FIGURE 7:**
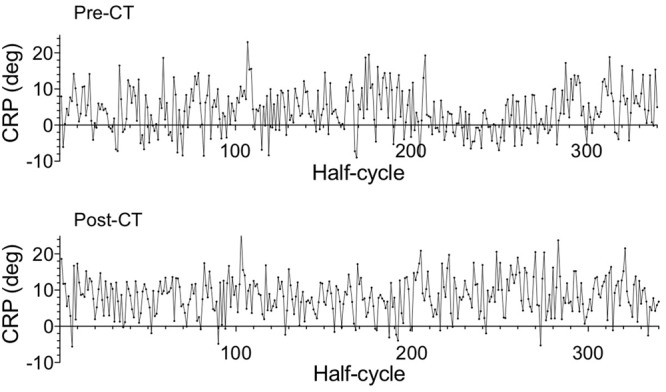
Average continuous relative phase (CRP) of the rowers’ oar movements for the 340 drive phases of the catch-finish half-cycles of the pre-CT (Upper) and the post-CT (Lower) sessions.

At the collective level, the RMS difference between oar positions (**Figure 6**) decreased from the pre-CT session (4.95 ± 2.38°) to the post-CT session (2.23 ± 1.77°), *d* = 1.30, indicating an increase in space-time similarity of the oar movements of the two rowers. Perhaps surprisingly, the nature of the between-rower coordination appeared to change as average CRP (**Figure 7**) evolved from -0.30 ± 4.44° (pre-CT) to -4.09 ± 3.86° (post-CT), *d* = 0.91, suggesting the coming to the fore of a phase lag of the stroke with respect to the bow rower. Inspection of **Figure 2**, however, suggests that, contrary to what is generally assumed (de Brouwer et al., 2013; de Poel et al., 2016; R’Kiouak et al., 2016; Seifert et al., 2017), average CRP may not adequately capture the subtleties of the changes in between-rower coordination. The timing of the catch by both rowers, for instance, became more closely time-locked, with the stroke-bow difference changing from -0.072 ± 0.055 s pre-CT to 0.003 ± 0.039 s post-CT (*d* = 1.57). Such a change was not observed for the timing of the finish, with the stroke-bow difference being 0.065 ± 0.105 pre-CT and 0.064 ± 0.081 post-CT, *d* = 0.01. Thus, during the pre-CT session, the bow rower entered the water somewhat before the stroke rower and left the water somewhat after the stroke rower. Over practice this timing difference disappeared for the catch, with both rowers entering the water at the same time after training, but not for the finish (release). The timing of the catch was not the only aspect that changed over practice; the position of the oars (i.e., oar angles) at catch and finish also evolved. The stroke-bow difference in oar angle decreased for the catch, from 4.18 ± 1.67° pre-CT to 1.79 ± 1.39° post-CT (*d* = 1.56), while the stroke-bow difference in oar angle increased for the finish, from 4.67 ± 1.36° pre-CT to 7.34 ± 0.90° post CT (*d* = 2.32). As can be seen from **Figure 2**, these results indicated that over practice the two rowers came to adopt similar oar angles when entering the water, while accentuating their difference (with a larger maximal angle for the stroke rower) when leaving the water.

Overall, changes at the collective level may thus be characterized as follows. Compared to the pre-CT session, during the post-CT session the rowers demonstrated a tighter synchronization and a closer correspondence in oar angle at the moment of catch. They also more closely matched the evolution over time of their subsequent oar movements, as indicated by the RMS oar-angle difference results. Ending the movement later than the bow rower, the stroke rower continued his oar movement up to a larger amplitude. The apparent lag of the stroke rower, as indicated by the average CRP results, is in fact the result of this pattern of coordination.

## Discussion

The purpose of the present study was to characterize how extensive training practice on a real-life performance-oriented joint-action task affected behavior at the collective and individual-agent levels. To this end we examined how the collective and individual oar behaviors of a newly formed pair of rowers evolved in sweep-oar rowing as a coxless pair over a one-and-a-half-month intensive crew training program. With both participating rowers being recognized individual sculling experts, the study allowed to focus on behavioral changes related to adaptation to the new task, without such changes being superseded by learning effects at the level of individual oar-handling capabilities.

At the scale of the collective crew behavior, results first of all indicated an overall increase in the space-time similarity of individual rowing patterns, as revealed by the decrease in RMS oar angle differences. This was to a large extent due to the catch points (marking the onset of the blades’ entry into the water) becoming more tightly matched between the two rowers in terms of both timing and oar-angle magnitude. However, the results also suggested a subtler change in the nature of the coordination of the rowers’ oar movements, with the stroke rower, developing an oar movement of a longer duration, continuing up to a larger amplitude than the bow rower in the post-CT session. This finding might be interpreted as the crew’s solution to (partially) avoid channeling the boat into yawing during the drive phase: full space-time similarity of the rowers’ oar movements in the asymmetrically rigged sweep-boat would indeed result in differences in the moments produced by each rower (Barrow, 2010). We note that —at least for sweep-boat rowing— the above-described particularities of the observed coordination pattern render the often-used average CRP measure (e.g., de Brouwer et al., 2013; de Poel et al., 2016; R’Kiouak et al., 2016; Seifert et al., 2017) rather ill-fitted to the job of comprehensively (and comprehensibility) capturing a rowing crew’s coordination pattern. This remains true, even when adopting a calculation method suitable for analysis of the water phase (catch-to-finish) half-cycles, as detailed in the Section “Materials and Methods” of this contribution. While at the moment of catch Relative Phase (RP) in the post-CT session was on average in fact very close to 0° (since the average catch time difference was a mere 0.003 s), the between-rower differences in duration and amplitude of oar movement resulted in average CRP values of -0.3° and -4.1° for the drive phases of pre-CT and post-CT sessions, respectively. Interpreting the latter as indicating that, overall, during the post-CT session the stroke lagged the bow rower (or, alternatively, that the bow led the stroke rower) would clearly not do justice to the subtleties of the coordination pattern observed. From the observation that during the post-CT session RP ≈ 0 at the moment of catch, we conclude that the crew studied did not appear develop a leader-follower relation (cf. Seifert et al., 2017). As illustrated in **Figure 2**, the two rowers rather performed in almost perfect harmony until the very end of the drive, with the stroke rower continuing his oar movement for a short time after the bow rower’s had ended.

The foregoing discussion already brings out that results observed at the level of the individual rowers consolidated and enlightened the idea that improvement in crew behavior was rooted in changes in how each rower performed his own movement. Interestingly, apart from the results on oar movement amplitude and timing alluded to above, we also observed training effects at the level of the *variability* of the kinematic patterns of oar movement (oar angle, OAV, and linear OWV). On the post-CT session the stroke rower demonstrated increased consistency (i.e., lower inter-cycle variability) over the drive phase on all three measures. While the bow rower also improved his consistency for OAV and linear OWV, he revealed an increase in variability in oar angle displacement during the drive phase.

The finding that, at the level of oar angular displacement, crew training resulted in a decrease of inter-cycle variability for one rower and an increase for the other rower is particularly noteworthy, as it speaks to the adaptability of individual patterns. Following training, the (initially more variable) stroke rower performed in a more stable manner, while the bow rower relaxed his initial tendency to always perform in the same way. Interestingly, this result highlights how a team member can change his behavior in terms of reducing its absolute efficiency (i.e., self-deteriorating his rowing pattern by increasing its variability) in order to obtain benefits at the team scale. Although we do not have direct proof for this, we suggest that the bow rower became better coupled to the stroke rower, enhancing the process of reciprocal compensation (Mottet et al., 2001; Araújo and Davids, 2016) that supports adaptability in joint action.

Overall, the results of the present case study, addressing both the crew- and individual-levels of analysis, allow us to tentatively discuss what building a team might imply in terms of the adaptations required. Both rowers were able to change their own individual patterns when they were trained to row as a team. Our study suggests that rowing together not only called for finding an efficient timing relation (i.e., finding the *when* of each rower’s oar movement), but also required each rower to change the *how* of the rowing movement. The training effects observed here complement findings from other domains (such as industrial and organizational psychology, see for instance Gorman et al., 2010) indicating that team building relies, at least in part, on *interactions*. Moreover, introducing perturbations into established team functioning (e.g., by changing teammates, Gorman et al., 2006) was found to improve team adaptability to novel situations. Procedures for improving team performance may thus benefit from taking a process-oriented, interaction-based approach (Gorman et al., 2006) rather than limiting oneself to a shared knowledge-oriented approach (Cooke et al., 2000). Indeed, the changes observed in (the variability of) rowing behaviors in the present study suggest that improved team performance was grounded in changes in the intrinsic dynamics of the individual team members. One might even speculate that the coaches’ choice to place the initially hyper-consistent rower in the bow (rather than in the stroke) position, thereby ensuring that he continuously saw his partner, originated from the perceived need to make him more adaptive in order to be successful in crew rowing.

Such adaptation of each individual’s intrinsic dynamics over crew practice brings up the question to which extent individuals having rowed in a team would be able to rapidly recover their individual patterns of rowing when performing alone anew (i.e., in their individual sculling practice). Oullier et al. (2008) reported that, after having been influenced by a sustained interaction, individual agents did not immediately return to their own intrinsic movement pattern, a phenomenon they referred to as social memory. Recently, Masumoto and Inui (2017) reported that in a joint motor action practicing together was important to enhance interaction capabilities of individual participants, and that retention effects were observable not only from individual practice to team performance, but also from team practice to individual performance. The question whether such effects may also exist in sport-specific practices opens promising directions for future research.

## Conclusion

We described how crew rowing changed over training and the extent to which it was associated with related changes in individual behaviors. Among the key results, our case study suggested the capability to change individual patterns of rowing as being the key element underlying the positive collective behavioral transformation. Our results also proposed individual variability of behavior as being an important variable to consider, while the need to either decrease or increase it to obtain team benefits may be task-dependent. In terms of the questions that remain open, the extent to which individual signatures converge or diverge through team training (and how it depends on initial (dis)similarities) should be further investigated in future research.

## Author Contributions

MF, MR, and JB conceived, designed and ran the study. MF, MR, RB, and JB analyzed and interpreted the data. All authors participated drafting the work and/or revising it critically for important intellectual content. The final version submitted was approved by all authors.

## Conflict of Interest Statement

The authors declare that the research was conducted in the absence of any commercial or financial relationships that could be construed as a potential conflict of interest. The reviewer RG and handling Editor declared their shared affiliation, and the handling Editor states that the process nevertheless met the standards of a fair and objective review.
